# Draft Genome Sequence of *Paenibacillus* sp. Strain DMB5, Acclimatized and Enriched for Catabolizing Anthropogenic Compounds

**DOI:** 10.1128/genomeA.00211-16

**Published:** 2016-03-31

**Authors:** Jenny Johnson, Binal Shah, Kunal Jain, Nidhi Parmar, Ankit Hinsu, Namrata Patel, Chaitanya G. Joshi, Datta Madamwar

**Affiliations:** a Environmental Genomics and Proteomics Laboratory, UGC Centre of Advanced Study, PG Department of Biosciences, Sardar Patel University, Bakrol, Anand, Gujarat, India; b Department of Animal Biotechnology, College of Veterinary Science and Animal Husbandry, Anand Agricultural University, Anand, Gujarat, India

## Abstract

Here, we present the draft genome sequence of *Paenibacillus* sp. strain DMB5, isolated from polluted sediments of the Kharicut Canal, Vatva, India, having a genome size of 7.5 Mbp and 7,077 coding sequences. The genome of this dye-degrading bacterium provides valuable information on the microbe-mediated biodegradation of anthropogenic compounds.

## GENOME ANNOUNCEMENT

Industrialization has become inevitable for socioeconomic development, but on the other hand, unacceptable industrial waste discharges impose a severe threat to civilization itself. The emerging level of pollution has accelerated the eradication of various natural resources of our planet Earth. Many contaminated vicinities are partially bioremediated by the natural attenuation processes, emphasizing the significance of the indigenous microbial population. Microbes that are catabolically diverse and flexible can endure the extremely varied conditions by inherently developing adaptive mechanisms. The exploitation of this microbial richness will enable us to explore the taxonomical mutualism and functional stability that prevail to allow microbial assemblages to coexist in such niches. Henceforth, we have distinguished a potent bacterium, *Paenibacillus* sp. strain DMB5, from the polluted sediments of Kharicut Canal, receiving toxic industrial effluents from the Vatva Industrial Estate, India. The bacterial isolate, in synergism with other bacteria, effectively degraded and metabolized azo dyes.

The genome of *Paenibacillus* sp. strain DMB5 was shotgun sequenced on an Ion Torrent platform using 318 Chip and 300-bp chemistry. The sequencing conferred 5,390,184 reads and 1.6 Gb of nucleotide bases, with an average read length of 312 bp. After quality filtering using PrinSeq-lite version 0.20.4 (1), the filtered reads were *de novo* assembled using the CLC Genomics Workbench software (CLC bio-Qiagen, Aarhus, Denmark), MIRA (2), and GS *de novo* assembler version 2.6. All three assemblies were then converged in CISA (3) to generate 174 contigs with a mean size of 42,857 bp and maximum length of 399,150 bp. The total genome size is 7,457,220 bp, with a G+C content of 51.2%.

The draft genome of *Paenibacillus* sp. DMB5 was annotated using the Rapid Annotations using Subsystems Technology (RAST) server (4) and the NCBI Prokaryotic Genome Annotation Pipeline (PGAP) (http://www.ncbi.nlm.nih.gov/genome/annotation_prok). The annotations revealed 7,077 coding sequences, 89 tRNA genes, and 17 rRNA genes. Various subsystems of essential functions, like carbohydrate metabolism, amino acid and derivatives, protein metabolism, etc., were discovered using RAST. Remarkably, the presence of several significant genes/operons in the *Paenibacillus* sp. DMB5 genome were observed that included the genes pertaining to resistance to antibiotics and toxic compounds, such as genes encoding metal efflux pump proteins for heavy metals, like mercury, chromium, arsenic, copper, cadmium, and zinc. Many stress-related proteins were identified as being related to oxidative stress, heat shock, and detoxification. Within the oxidative stress category, NADH quinone oxidoreductases, monooxygenases, and dioxygenases were also annotated that are reported to catalyze the reduction of azo dyes (5–7). Moreover, a number of proteins involved in various aromatic compound degradation pathways were noticed, including pathways for quinate, salicylate, and gentisate degradation. The relevance of these catabolic genes in the draft genome validates the potential of *Paenibacillus* sp. DMB5 for the biodegradation of xenobiotic compounds. However, the pursuit to explore the underlying degradation mechanisms and biochemical pathways in strain DMB5 still remains.

### Nucleotide sequence accession numbers.

This whole-genome shotgun project has been deposited at DDBJ/EMBL/GenBank under the accession no. LRAC00000000. The version described in this paper is version LRAC01000000.
